# Application value of different imaging methods in the early diagnosis of small hepatocellular carcinoma: a network meta-analysis

**DOI:** 10.3389/fonc.2024.1510296

**Published:** 2025-01-14

**Authors:** Jian Dong, Zhen Wang, Si-Rui Wang, Huan Zhao, Jun Li, Ting Ma

**Affiliations:** Department of Ultrasound Medicine, the First Affiliated Hospital of Shihezi University, Shihezi, Xinjiang, China

**Keywords:** diagnostic imaging, small hepatocellular carcinoma, multiple diagnostic methods, network meta-analysis, ultrasound

## Abstract

**Objective:**

To determine the diagnostic value of ultrasound, multi-phase enhanced computed tomography, and magnetic resonance imaging of small hepatocellular carcinoma.

**Methods:**

Experimental studies on diagnosing small hepatocellular carcinoma in four databases: PubMed, Cochrane Library, Web of Science, and Embase, were comprehensively searched from October 2007 to October 2024. Relevant diagnostic accuracy data were extracted and a Bayesian model that combined direct and indirect evidence was used for analysis.

**Results:**

16 original studies were included and data from 2,447 patients were collated to assess the diagnostic value of 10 different methods. The methodological quality of the included studies was good and there was no obvious publication bias. The pooled DOR of all diagnostic methods was 19.61, which was statistically significant (I^2^ = 76.0%, *P* < 0.01, 95% CI:13.30 - 28.92). Normal US + CEUS + ultrasonic elastic imaging had the highest specificity (92.9), accuracy (93.6), and positive predictive value (94.4). Unenhanced MRI + Contrast-enhanced MRI had the highest sensitivity (96.6) and negative predictive value (96.6), but specificity (12.5) and positive predictive value (34.4) were extremely poor. Contrast-enhanced MRI had the highest diagnostic value in individual imaging methods (sensitivity: 66, specificity: 55.5, accuracy: 67.9, positive predictive value: 64.4, negative predictive value: 66.5). There was significant inconsistency and high heterogeneity in this study.

**Systematic review registration:**

https://www.crd.york.ac.uk/PROSPERO/, identifier CRD42024507883.

## Introduction

Hepatocellular carcinoma (HCC) is one of the responsible for a significant number of cancer-related deaths globally (1). HCC is characterized by atypical symptoms in its early stages and distant metastases in its late stages, which significantly impacts the patient’s prognosis (2). Small hepatocellular carcinoma(sHCC) is a crucial stage in the initial development and progression of hepatocellular carcinoma. sHCC usually has not yet developed distant metastasis. Compared to ordinary HCC patients who do not belong to sHCC, patients have better overall conditions, with the possibility of achieving a better prognosis through treatment methods. Currently, surgical treatment remains the standard treatment for sHCC. The main surgical procedures include radiofrequency ablation (RFA), surgical resection (SR), trans-arterial chemoembolization, and so on (3, 4). Carcinomas below 3.0 cm are considered to have the potential to be completely cured by RFA treatment, whereas HCC growing beyond the critical size becomes more aggressive and leads to worse clinical outcomes (5). For sHCC patients with a tumor diameter less than 3.0 cm and AFP < 200 ng/mL, the treatment modality did not show any significant association with patients’ overall survival or disease-free survival, but ordinary HCC patients who do not belong to sHCC usually achieve non-satisfactory treatment outcomes (6, 7). The complication rate of RFA in sHCC is 2%-7.9% and the mortality rate is 0%-1.6%. In contrast, ordinary HCC patients who do not belong to sHCC typically require SR treatment, with a complication rate of 27.5% (8–14). Early screening of tumors and implementing RFA treatment during the sHCC stage may lead to optimal treatment outcomes and fewer adverse consequences. Ordinary HCC patients who do not belong to sHCC may experience late-stage distant metastasis and lose the opportunity for surgery, requiring palliative radiotherapy treatment. Their quality of life and prognosis are extremely poor. Finally, for some tumors with good location, RFA and SR have a similar recurrence rate or overall survival rate, but tumors located in the subphrenic or perivascular area have a higher recurrence rate of RFA. Therefore, early and accurate screening and accurate analysis of tumor anatomical location play a crucial role in guiding the clinical determination of surgical plans, early decision-making of treatment, and improving patient quality of life.

The field of imaging has been advancing rapidly. The clinical diagnostic value of typical imaging manifestations has significantly surpassed that of the reference standard for malignant tumor diagnosis: histopathological examination, in the confirmation of the diagnosis in patients with highly suspected HCC (15, 16). The commonly used imaging diagnostic methods for HCC in clinical practice currently include: ultrasound (US), multi-phase enhanced computed tomography (MDCT), magnetic resonance imaging (MRI), digital subtraction angiography, and positron emission tomography (17–20). The diagnostic criteria for sHCC, which is characterized by small size, partially hyperemic blood supply, and an imaging phenotype that is easily confused with high-grade dysplastic nodule (21, 22), has still not formed a unified consensus, which has seriously affected the progress of clinical therapeutic measures and the prolongation of the overall survival of patients (23). Due to the lack of specificity of the typical imaging manifestations of sHCC, diagnosis by individual imaging methods may be difficult. Whether the combined diagnosis of two or more imaging methods can improve the diagnostic accuracy of sHCC remains to be evaluated more thoroughly.

Therefore, to evaluate the diagnostic accuracy of screening for sHCC smaller than 3.0cm through imaging methods in daily clinical practice. We conducted a meta-analysis of different imaging methods to diagnose sHCC and evaluate their performance and efficacy. We retrospectively included data from the literature on diagnostic experiments of sHCC by commonly used imaging methods in clinical practice, such as US, MDCT, and MRI, in the individual or combined diagnosis of sHCC. We conducted a multifaceted analysis of available diagnostic evidence to evaluate the diagnostic value of various diagnostic methods for sHCC. and to determine the most effective diagnostic imaging methods. The evidence for decision-making was synthesized to provide a diagnostic basis for physicians when making clinical decisions.

## Materials and methods

### Retrieval strategies

We comprehensively searched four databases: PubMed, Embase, Web of Science, and Cochrane Library. The search terms were “small hepatocellular carcinoma”, “diagnosis”, “ultrasound contrast”, “elasticity imaging techniques”, “magnetic resonance imaging”, “multi-phase enhanced computed tomography”, “ultrasonography”, “Positron emission tomography CT”, and “18F-fluorodeoxyglucose imaging”, etc. We conducted a thorough manual search of the literature. Due to the continuous development of imaging methods for sHCC, we set the time frame for searching the literature from 2007 to 2024. At the same time, we did not restrict the language of the studies except Chinese to ensure a complete literature search. Based on the above principles, we also searched the references of the relevant literature simultaneously.

### Inclusion criteria

The following aspects are considered as the inclusion criteria for this study (1): patients with imaging findings showing the presence of isolated nodules with a diameter less than 3cm in the liver (2); simultaneous comparison of two or more diagnostic imaging methods for sHCC (3); having a clear diagnostic reference standard, and surgery, biopsy, combination with imaging and laboratory indicators are all allowed (4). true positive (TP), false positive (FP), false negative (FN), and true negative (TN) data can be collected either directly or indirectly, and (5) the study design used in the article was a diagnostic pilot study.

### Exclusion criteria

The following aspects are considered as the exclusion criteria for this study (1): case reports, editorial comments, letters to the editor, and lecture literature (2); no clear inclusion and exclusion criteria in the study (3); incomplete diagnostic data for research (4); the research content is not related to sHCC, or there are patients with recurrent HCC or metastatic cancer present; and (5) literature not available in full text.

### Literature screening

Two researchers screened the titles and abstracts of studies retrieved using the search strategy and from other sources. The aim was to identify diagnostic trial studies that met the inclusion and exclusion criteria. The selected papers thoroughly reviewed the full text to determine eligibility for inclusion in the analysis. A third-party senior review investigator was consulted to make the final decision in case of any disagreement.

### Data collection

The researcher carefully read the full text of the literature, created a standardized database extraction form, and extracted the year of publication of the included studies, with a focus on obtaining the data of TP, FP, FN, and TN diagnostic tests. For the literature where multiple guideline diagnostic data existed, the data of Liver Imaging Reporting and Data System was collected by our research group. When multiple Observer data existed, we performed averaging.

### Methodological quality assessment

Two authors used the Quality Assessment of Diagnostic Accuracy Studies-2 (QUADAS-2) scale to evaluate the methodological quality of the diagnostic experiments included (24). To prevent investigators’ subjective biases from affecting the evaluation of a study’s risk of bias, any disagreements between two authors regarding the risk of bias in a specific study were resolved through discussion. If necessary, a third-party senior review investigator was also involved in the discussion.

### Statistical analysis

We conducted a network meta-analysis by categorizing various diagnostic imaging methods for sHCC to accurately judge the accuracy of each imaging method in diagnosing a condition. Plotting was performed by extracting data from the literature on diagnostic performance to construct a 2*2 league table of TP, FP, FN, and TN, and the data on sensitivity (SEN), specificity (SPE), accuracy (ACC), positive predictive value (PPV), and negative predictive value (NPV) of each diagnostic study were calculated. Diagnostic Ratio (DOR) is the odds of a positive test result in patients with the disease compared to those without (25). In this study, the relative diagnostic efficiency of each imaging method was assessed by detecting the DOR. Network diagrams were generated using Stata V.18.0 software (Silicon Valley, America) to directly present the comparative results of each imaging method. In the network diagrams, each node represented an imaging diagnostic method and the thickness of the connecting line between nodes represents the number of studies comparing two diagnostic methods (26). The inconsistency test is a test to verify that the statistical test satisfies the assumptions of homogeneity, transmissibility, and consistency of the network meta-analysis. Global inconsistency assumptions were made using a node-splitting method. When the *P*-value is greater than 0.05, it demonstrates that the overall consistency assumption can be accepted for each diagnostic method. Local inconsistency was assessed and both direct and indirect comparisons were evaluated for consistency. The consistency assumption was only acceptable if no inconsistency was found in both global and local inconsistency test tests (27). For data with possible inconsistencies we performed loop inconsistency tests to discuss the need for a Cochran Q test for heterogeneity to detect differences between loops. To evaluate the heterogeneity between studies, the Cochran Q statistic and the I^2^ index were utilized. Significant heterogeneity was indicated when the *P* value of the Cochran Q chi-square test was lower than 0.05 and/or the I^2^ statistic exceeded 50%. The overall magnitude of comparative effectiveness between interventions was illustrated by producing a forest plot (26). Diagnostic performance can be determined by ranking diagnostic effect sizes in ascending or descending order using the area under the cumulative ranking curve (SUCRA) (28). To evaluate the presence of publication bias in the study, a funnel plot was created. Sensitivity Analyses can be performed by excluding low-quality studies one by one if necessary, re-estimating the combined effect size and observing whether the combined results have changed significantly by comparing them with the results of the study before the exclusion, to determine the reasons for differences and evaluate the reliability of the calculations (29, 30).

## Results

### Study of baseline characteristics

**Figure 1:**
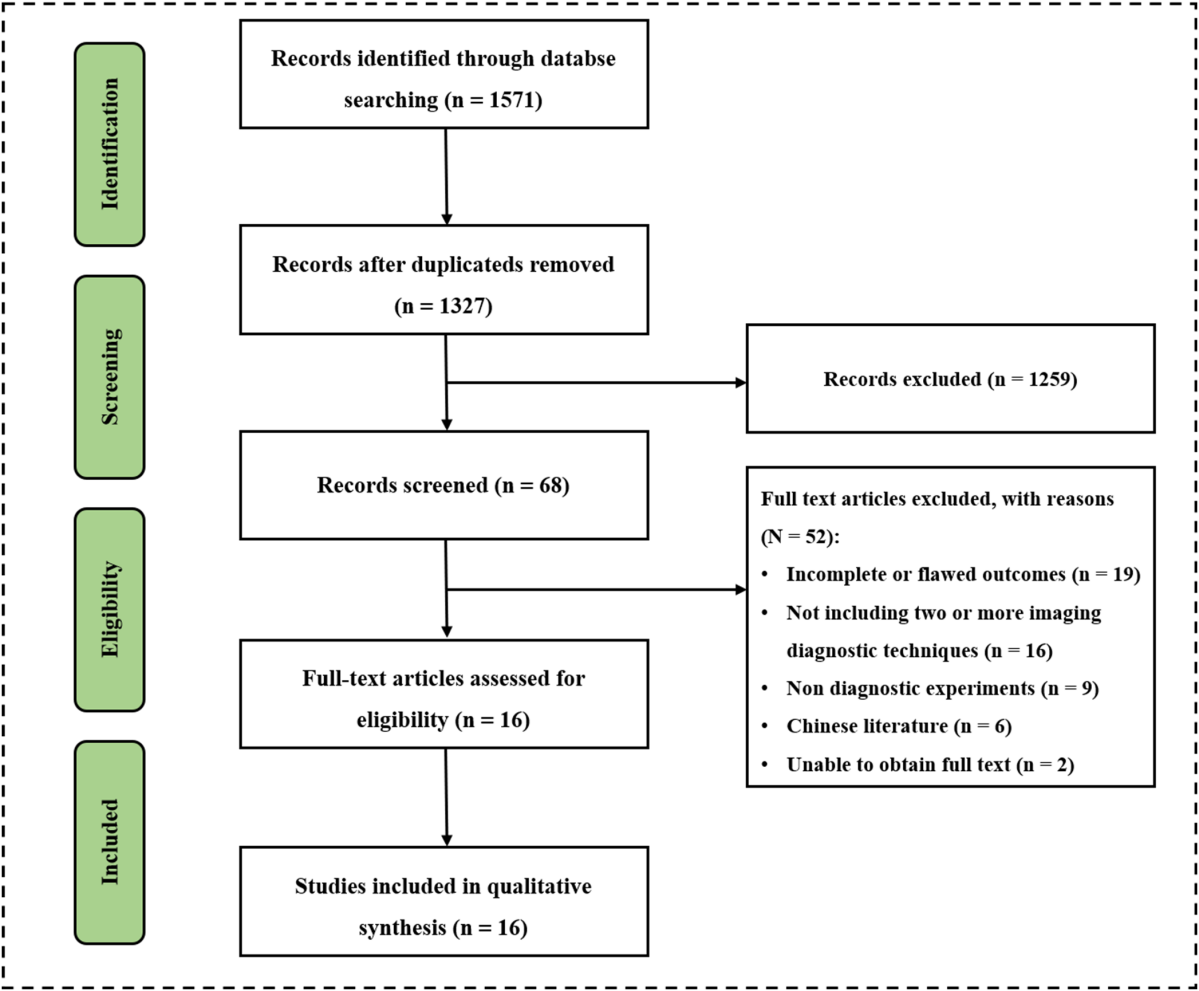
Flow chart of literature retrieval and sereening.

The researchers comprehensively searched 1571 potentially relevant documents from four databases. After excluding 224 duplicate files, there were 1327 remaining. After conducting a preliminary search and reviewing the titles and abstracts of 1327 papers, two researchers found 68 papers suitable for their research purpose. Two papers could not be accessed in full text so two researchers read the full text of 66 papers. The study analyzed and screened sixteen papers and 2447 patients strictly according to the inclusion and exclusion criteria for inclusion (**Figure 1**) (30–46). There were twelve prospective studies and four retrospective studies. Nine papers used US for diagnosis, including three using normal US, nine using contrast-enhanced ultrasound (CEUS), and one using ultrasonic elastic imaging; Eight papers used MDCT for diagnosis; thirteen papers used MRI for diagnosis, including four using Unenhanced MRI and eleven using Enhanced MRI; Six papers used two or more methods for diagnosis, including four using MDCT + Unenhanced MRI, one using Unenhanced MRI + Contrast-enhanced MRI, and one using Normal US + CEUS + ultrasonic elastic imaging. Baseline characteristics such as year of publication, authors, country, basic information of included patients, diagnostic imaging methods used, and reference standard of the included studies were extracted and summarized in **Supplemental Table 1**.

### Methodological quality assessment

The methodological quality of the 16 included diagnostic trials was assessed by the QUADAS-2 scale, and the included studies showed high quality (**Supplementary Figure 1**). Five of them were deemed to have a high risk of bias in the study. Masatoshi Kudo et al. (35) had neither a temporal threshold for inclusion of the patient inclusion process nor an indication of whether the patient inclusion was continuous or randomized, and therefore its patient selection was deemed to be high risk. There were cases in which some patients received different reference standards in the evaluation of flow and timing in the study by A. Granito et al. (34) Masatoshi Kudo et al. (35) François Le Moigne et al. (36) Maxime Ronot et al. (38) and Hye Young Sun et al. (41) which may have been subject to validation bias its reference standards were deemed to be high risk. None of the remaining eleven papers showed a significant risk of bias, and the overall methodological quality was evaluated as good.

### Risk of bias

**Figure 2:**
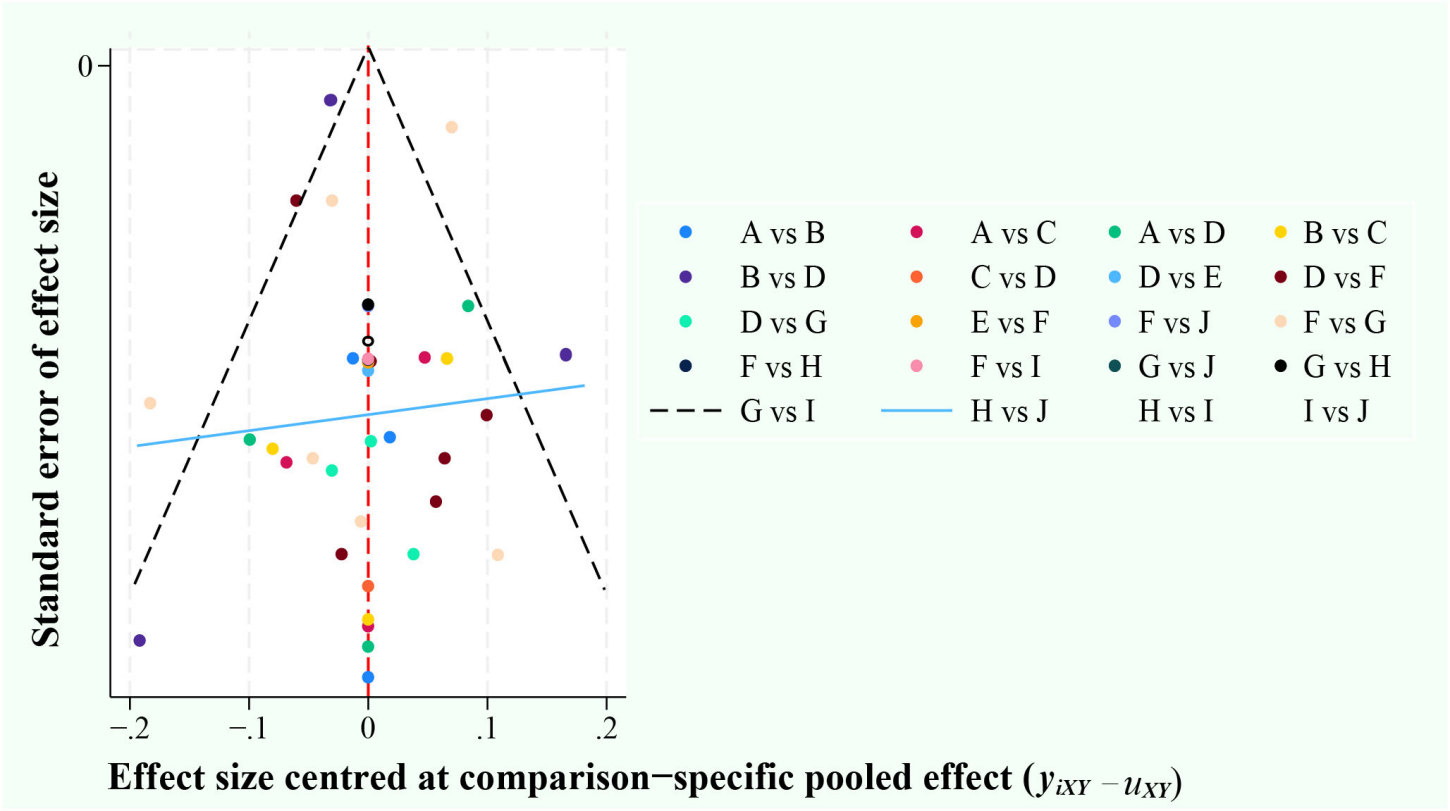
Funnel plots. A, ultrasonic elastic imaging; B, Normal US; C, Normal US + CEUS + ultrasonic elastic imaging; D, CEUS; E, CEUS + Contrast-enhanced MRI; F, Contrast-enhanced MRI; G, MDCT; H, Unenhanced MRI; I, MDCT + Unenhanced MRI; J, Unenhanced MRI + Contrast-enhanced MRI.

The risk of bias in this literature was determined by constructing network funnel plots of the 10 diagnostic methods. The results showed good symmetry, and no significant risk of bias was observed in the literature that included this. (**Figure 2**).

### Network diagrams

**Figure 3:**
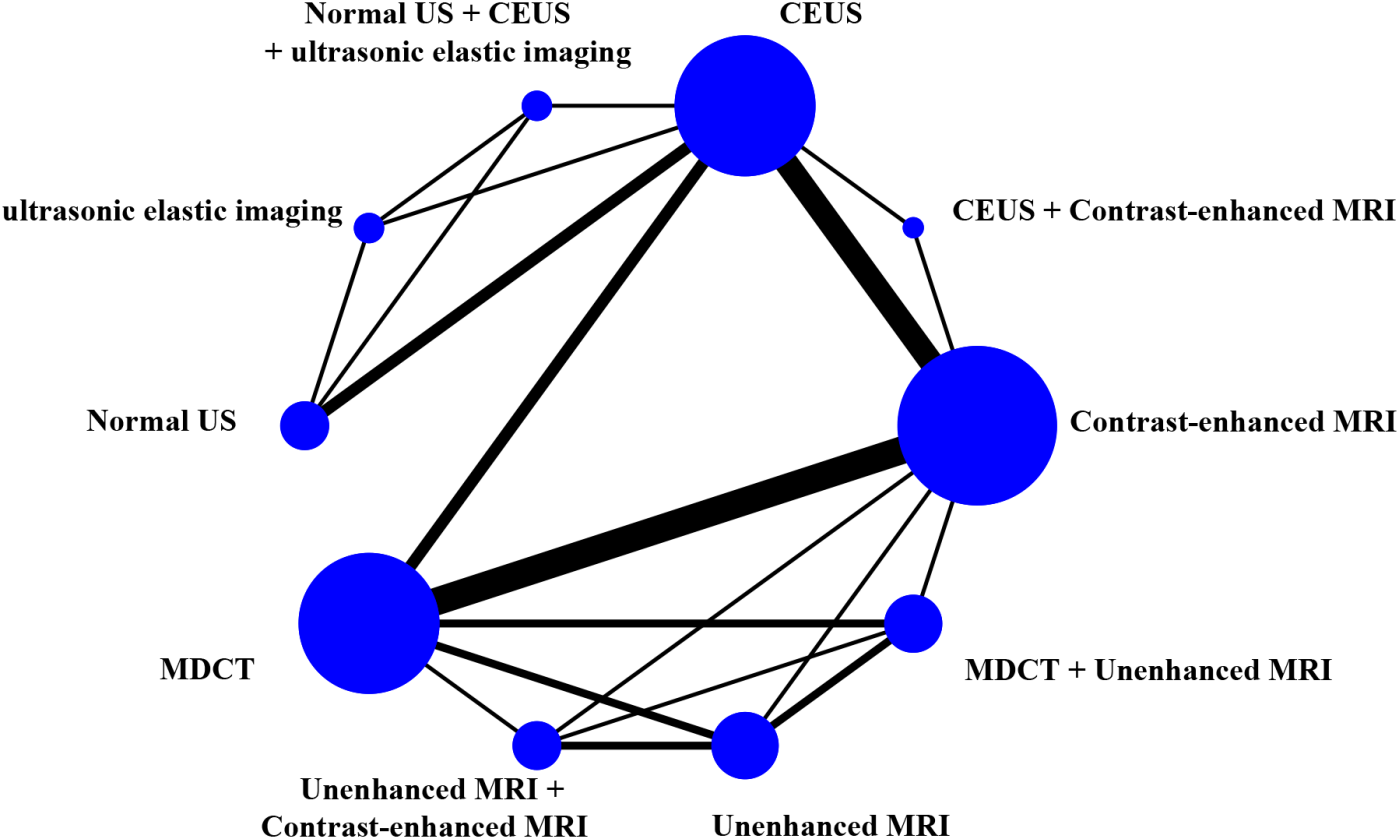
Network diagrams.

The study generated network diagrams to directly present the results of comparing the imaging methods (**Figure 3**). It was found that the three diagnostic methods: CEUS, Contrast-enhanced MRI, and MDCT, had larger nodes and included larger sample sizes; the CEUS + Contrast-enhanced MRI experimental diagnostic method had the smallest nodes and included the smallest sample sizes. Direct comparisons between diagnostic methods interacted well, with the thickest connecting segments between Contrast-enhanced MRI and MDCT, and the most studies participating in the comparison.

### Inconsistency test

The study conducted an inconsistency test of the included literature through Stata V.18.0 software (Silicon Valley, America). The results of the SEN inconsistency test were found to be significant (*P* = 0.04). A forced consistency test found Unenhanced MRI + Contrast-enhanced MRI to be significant compared to the control model, with possible inconsistency between the two (*P* = 0.02, 95% CI: 0.09 - 0.90). All results were greater than 0.05 when local inconsistency tests were performed, and when loop inconsistency tests were performed. There were partial loop arms with 95% confidence intervals that did not cross the zero-effect line (**Supplementary Figure 2**). Overall, there was significant inconsistency in the SEN.

The SPE inconsistency test was significant (*P* < 0.01). All of the outcomes of the test for local inconsistency had a value greater than 0.05, and when loop inconsistency tests were performed, there were partial loop arms with 95% confidence intervals that did not cross the zero-effect line (**Supplementary Figure 3**). Significant inconsistency was present in the SPE.

The results found that the ACC inconsistency test, the PPV inconsistency test, and the NPV inconsistency test were all not significant (ACC: *P* = 0.85, PPV: *P* = 0.21, NPV: *P* = 0.74).

### SUCRA sequencing of sHCC diagnostic methods

The study used SUCRA to rank the diagnostic effects of sHCC to determine diagnostic performance. The diagnostic measures with higher rankings correspond to larger SUCRA values (47). The SUCRA results of this study are summarized in **Table 1**. It can be found that Unenhanced MRI + Contrast-enhanced MRI diagnosis exhibited the highest SUCRA values in both SEN and NPV (SEN: 96.6, NPV: 96.6). The diagnostic method that presented the best SUCRA values in SPE, ACC, and PPV was Normal US + CEUS + ultrasonic elastic imaging (SPE: 92.9, ACC: 93.6, PPV: 94.4). Among the individual imaging methods, the method with overall higher SUCRA was Contrast-enhanced MRI (SEN: 66.0, SPE: 55.5, ACC: 67.9, PPV: 64.4, NPV: 65.5).

**Table 1:** SUCRA ranking of sHCC diagnostic methods.

DIAGNOSTIC METHODS	SUCRA values				
	SEN	SPE	ACC	PPV	NPV
Ultrasonic elastic imaging	20.7	64.7	33.2	47.8	35.4
Normal US	2.6	62.1	9.4	17.6	12.3
Normal US + CEUS + ultrasonic elastic imaging	64.2	92.9	93.6	94.4	91.7
CEUS	34.5	61.6	32.6	63.2	33.6
CEUS + Contrast-enhanced MRI	28.3	73.8	24.9	81.6	19.2
Contrast-enhanced MRI	66	55.5	67.9	64.4	66.5
MDCT	41.4	47.9	38.4	43.3	38.2
Unenhanced MRI	80.3	21.6	70.5	41.9	73.3
MDCT + Unenhanced MRI	65.5	7.5	40.5	11.4	33.2
Unenhanced MRI + Contrast-enhanced MRI	96.6	12.5	89.1	34.4	96.6

The number with the background color indicates the highest SUCRA;

SUCRA, the area under the cumulative ranking curve; US, ultrasound; CEUS, Contrast-enhanced ultrasound; MRI, magnetic resonance imaging; and MDCT, multi-phasic enhanced computed tomography.

### Diagnostic ratio

**Figure 4:**
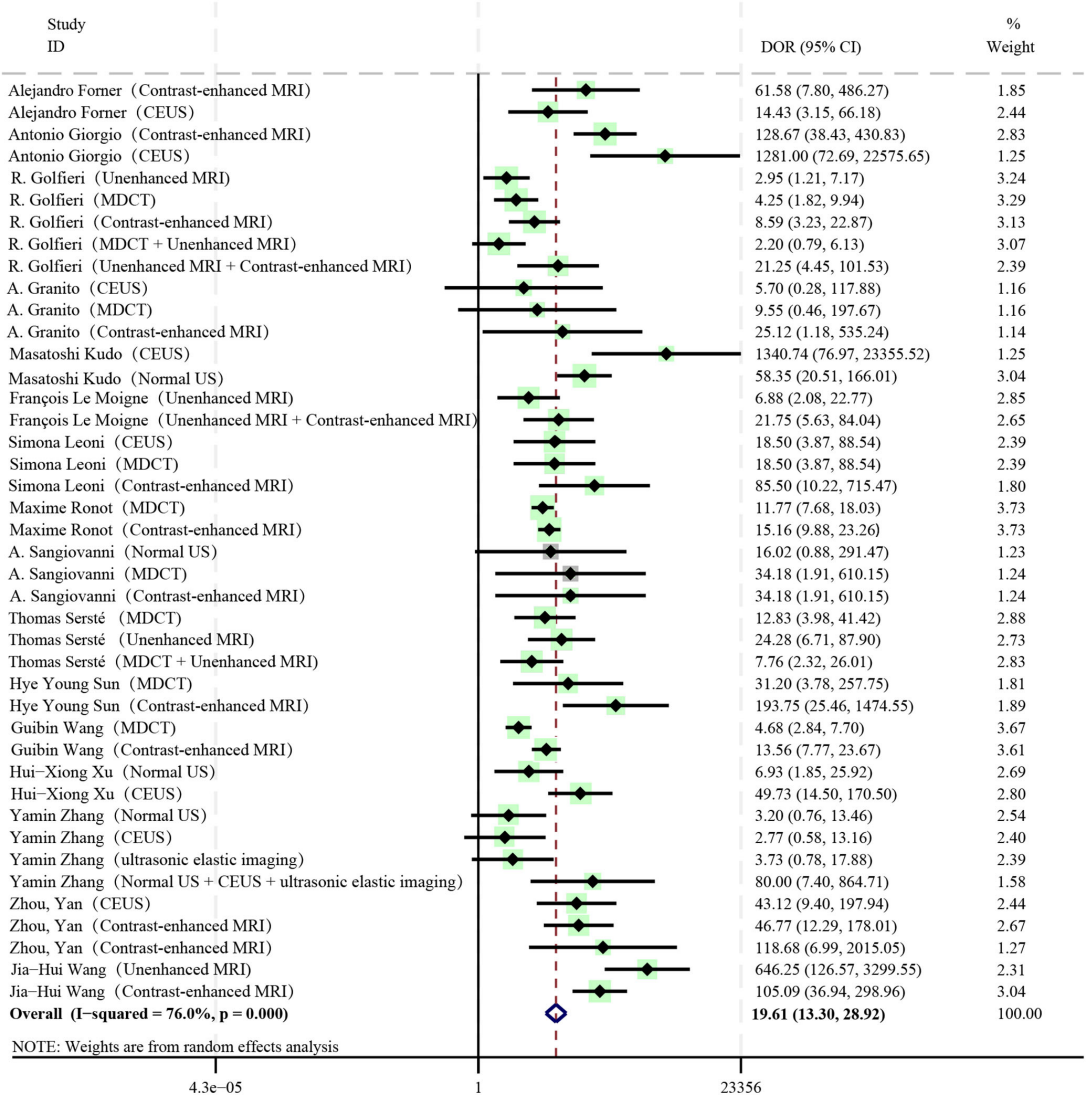
Diagnostic ratio.

The pooled DOR of all diagnostic methods included in the study was 19.61, which was statistically significant (I^2^ = 76%, *P* < 0.01, 95%CI: 13.30 - 28.92) (**Figure 4**). Among them, Contrast-enhanced MRI (DOR: 27.42, 95%CI: 15.44 - 48.72), Unenhanced MRI + Contrast-enhanced MRI (DOR: 21.53, 95%CI: 7.74 - 59.88), Normal US + CEUS + ultrasonic elastic imaging (DOR: 80.00, 95%CI: 7.40 - 864.71) all had relatively high DOR values.

### Normal US + CEUS + ultrasonic elastic imaging

Combined ultrasound diagnostic methods demonstrated very high diagnostic efficacy in a two-by-two comparison. The pooled SEN, SPE, ACC, PPV, and NPV of Normal US + CEUS + ultrasonic elastic imaging for diagnosing sHCC were 64.2, 92.9, 93.6, 94.4, and 91.7 respectively. Compared with Unenhanced MRI (OR: 0.46, 95%CI: 0.06 - 0.87), and MDCT + Unenhanced MRI (OR: 0.58, 95%CI: 0.15 - 1.00), Normal US + CEUS + ultrasonic elastic imaging demonstrated a higher SPE (**Table 2**). Compared with MDCT (OR: 0.22, 95%CI: 0.01 - 0.43), ultrasonic elastic imaging (OR: 0.26, 95%CI: 0.03 - 0.48), CEUS (OR: 0.24, 95%CI: 0.05 - 0.43), CEUS + Contrast - enhanced MRI (OR: 0.27, 95%CI: 0.03 - 0.51), and Normal US (OR: 0.32, 95%CI: 0.13 - 0.51), Normal US + CEUS + ultrasonic elastic imaging demonstrated higher ACC (**Table 3**). Compared with MDCT + Unenhanced MRI (OR: 0.18, 95%CI: 0.03 - 0.33), and Normal US (OR: 0.17, 95%CI: 0.04 - 0.29), Normal US + CEUS + ultrasonic elastic imaging demonstrated higher PPV (**Table 4**). The combined ultrasound diagnostic methods had a higher NPV compared with all methods except Unenhanced MRI (**Table 5**).

**Table 2:** League table on SPE.

Unenhanced MRI + Contrast-enhanced MRI	Normal US + CEUS + ultrasonic elastic imaging	Contrast-enhanced MRI	Unenhanced MRI	MDCT	Ultrasonic elastic imaging	CEUS	MDCT + Unenhanced MRI	CEUS + Contrast-enhanced MRI	Normal US
**Unenhanced MRI + Contrast-enhanced MRI**	0.53 (0.97,0.10)	0.25 (0.01,0.50)	0.07 (-0.16,0.29)	0.22 (-0.03,0.47)	0.32 (-0.13,0.77)	0.29 (-0.00,0.57)	-0.04 (-0.31,0.23)	0.36 (-0.02,0.74)	0.38 (0.06, 0.69)
**-0.53 (-0.97,-0.10)**	**Normal US + CEUS + ultrasonic elastic imaging**	-0.28 (-0.64,0.08)	-0.46 (-0.87,-0.06)	-0.31 (-0.69,0.06)	-0.21 (-0.57,0.15)	-0.25 (-0.57,0.08)	-0.58 (-1.00,-0.15)	-0.17 (-0.60,0.26)	-0.24 (-0.52, 0.05)
**-0.25 (-0.50,-0.01)**	0.28 (-0.08,0.64)	**Contrast-enhanced MRI**	-0.18 (-0.37,0.00)	-0.03 (-0.17,0.10)	0.07 (-0.31,0.45)	0.03 (-0.12,0.19)	-0.29 (-0.52,-0.07)	0.11 (-0.18,0.40)	0.04 (-0.18, 0.26)
-0.07 (-0.29,0.16)	**0.46 (0.06,0.87)**	**0.18 (-0.00,0.37)**	**Unenhanced MRI**	0.15 (-0.04,0.34)	0.26 (-0.16,0.67)	0.22 (-0.02,0.46)	-0.11 (-0.33,0.11)	0.29 (-0.05,0.64)	0.35 (0.06, 0.65)
-0.22 (-0.47,0.03)	0.31 (-0.06,0.69)	0.03 (-0.10,0.17)	-0.15 (-0.34,0.04)	**MDCT**	0.11 (-0.29,0.50)	0.07 (-0.13,0.26)	-0.26 (-0.48,-0.04)	0.14 (-0.17,0.46)	0.11 (-0.14, 0.35)
-0.32 (-0.77,0.13)	0.21 (-0.15,0.57)	-0.07 (-0.45,0.31)	-0.26 (-0.67,0.16)	-0.11 (-0.50,0.29)	**ultrasonic elastic imaging**	-0.04 (-0.38,0.31)	-0.37 (-0.80,0.07)	0.04 (-0.41,0.49)	-0.03 (-0.34, 0.28)
**-0.29 (-0.57,0.00)**	0.25 (-0.08,0.57)	-0.03 (-0.19,0.12)	-0.22 (-0.46,0.02)	-0.07 (-0.26,0.13)	0.04 (-0.31,0.38)	**CEUS**	-0.33 (-0.60,-0.06)	0.08 (-0.21,0.36)	0.01 (-0.16, 0.18)
0.04 (-0.23,0.31)	**0.58 (0.15,1.00)**	**0.29 (0.07,0.52)**	0.11 (-0.11,0.33)	**0.26 (0.04,0.48)**	0.37 (-0.07,0.80)	**0.33 (0.06,0.60)**	**MDCT + Unenhanced MRI**	0.40 (0.04,0.77)	0.41 (0.11, 0.70)
-0.36 (-0.74,0.02)	0.17 (-0.26,0.60)	-0.11 (-0.40,0.18)	-0.29 (-0.64,0.05)	-0.14 (-0.46,0.17)	-0.04 (-0.49,0.41)	-0.08 (-0.36,0.21)	**-0.40 (-0.77,-0.04)**	**CEUS + Contrast-enhanced MRI**	-0.07 (-0.37, 0.23)
-0.30 (-0.64,0.05)	0.24 (-0.09,0.56)	-0.04 (-0.29,0.21)	-0.23 (-0.54,0.08)	-0.08 (-0.35,0.20)	0.03 (-0.32,0.37)	-0.01 (-0.20,0.19)	**-0.34 (-0.67,-0.00)**	0.07 (-0.28,0.41)	**Normal US**

US, ultrasound; CEUS, Contrast-enhanced ultrasound; MRI, magnetic resonance imaging; and MDCT, multi-phasic enhanced computed tomography.

The form with the background color indicates the diagnostic method used in this article;

The numerical value represents the comparison of diagnostic efficiency between two diagnostic methods and bolded data represents a meaningful difference in diagnostic performance between the two diagnostic methods.

**Table 3:** League table on ACC.

Unenhanced MRI + Contrast-enhanced MRI	Normal US + CEUS + ultrasonic elastic imaging	Contrast-enhanced MRI	Unenhanced MRI	MDCT	ultrasonic elastic imaging	CEUS	MDCT + Unenhanced MRI	CEUS + Contrast-enhanced MRI	Normal US
**Unenhanced MRI + Contrast-enhanced MRI**	0.06 (-0.18,0.30)	-0.09 (-0.21,0.03)	-0.08 (-0.19,0.03)	-0.16 (-0.29,-0.04)	-0.20 (-0.46,0.07)	-0.18 (-0.32,-0.04)	-0.16 (-0.30,-0.02)	-0.21 (-0.40,-0.02)	-0.26 (-0.43,-0.09)
-0.06 (-0.30,0.18)	**Normal US + CEUS + ultrasonic elastic imaging**	-0.15 (-0.36,0.05)	-0.14 (-0.36,0.08)	-0.22 (-0.43,-0.01)	-0.26 (-0.48,-0.03)	-0.24 (-0.43,-0.05)	-0.22 (-0.45,0.02)	-0.27 (-0.51,-0.03)	-0.32 (-0.51,-0.13)
0.09 (-0.03,0.21)	0.15 (-0.05,0.36)	**Contrast-enhanced MRI**	0.01 (-0.08,0.10)	-0.07 (-0.13,-0.01)	-0.10 (-0.34,0.13)	-0.09 (-0.16,-0.01)	-0.06 (-0.18,0.06)	-0.12 (-0.27,0.03)	-0.17 (-0.29,-0.04)
0.08 (-0.03,0.19)	0.14 (-0.08,0.36)	-0.01 (-0.10,0.08)	**Unenhanced MRI**	-0.08 (-0.18,0.01)	-0.12 (-0.37,0.14)	-0.10 (-0.21,0.01)	-0.08 (-0.19,0.04)	-0.13 (-0.31,0.04)	-0.18 (-0.33,-0.03)
**0.16 (0.04,0.29)**	**0.22 (0.01,0.43)**	-0.15 (-0.36,0.05)	0.08 (-0.01,0.18)	**MDCT**	-0.03 (-0.28,0.21)	-0.02 (-0.10,0.07)	0.01 (-0.11,0.12)	-0.05 (-0.21,0.11)	-0.10 (-0.23,0.03)
0.20 (-0.07,0.46)	**0.26 (0.03,0.48)**	0.10 (-0.13,0.34)	0.12 (-0.14,0.37)	0.03 (-0.21,0.28)	**ultrasonic elastic imaging**	0.02 (-0.21,0.24)	0.04 (-0.23,0.30)	-0.02 (-0.29,0.26)	-0.06 (-0.29,0.16)
**0.18 (0.04,0.32)**	**0.24 (0.05,0.43)**	**0.09 (0.01,0.16)**	0.10 (-0.01,0.21)	0.02 (-0.07,0.10)	-0.02 (-0.24,0.21)	**CEUS**	0.02 (-0.11,0.16)	-0.03 (-0.18,0.12)	-0.08 (-0.18,0.02)
**0.16 (0.02,0.30)**	0.22 (-0.02,0.45)	0.06 (-0.06,0.18)	0.08 (-0.04,0.19)	-0.01 (-0.12,0.11)	-0.04 (-0.30,0.23)	-0.02 (-0.16,0.11)	**MDCT + Unenhanced MRI**	-0.06 (-0.25,0.13)	-0.10 (-0.27,0.07)
**0.21 (0.02,0.40)**	**0.27 (0.03,0.51)**	0.12 (-0.03,0.27)	0.13 (-0.04,0.31)	0.05 (-0.11,0.21)	0.02 (-0.26,0.29)	0.03 (-0.12,0.18)	0.06 (-0.13,0.25)	**CEUS + Contrast-enhanced MRI**	-0.05 (-0.23,0.13)
**0.26 (0.09,0.43)**	**0.32 (0.13,0.51)**	**0.17 (0.04,0.29)**	**0.18 (0.03,0.33)**	0.10 (-0.03,0.23)	0.06 (-0.16,0.29)	0.08 (-0.02,0.18)	0.10 (-0.07,0.27)	0.05 (-0.13,0.23)	**Normal US**

US, ultrasound; CEUS, Contrast-enhanced ultrasound; MRI, magnetic resonance imaging; and MDCT, multi-phasic enhanced computed tomography.

The form with the background color indicates the diagnostic method used in this article;

The numerical value represents the comparison of diagnostic efficiency between two diagnostic methods and bolded data represents a meaningful difference in diagnostic performance between the two diagnostic methods.

**Table 4:** League table on PPV.

Unenhanced MRI + Contrast-enhanced MRI	Normal US + CEUS + ultrasonic elastic imaging	Contrast-enhanced MRI	Unenhanced MRI	MDCT	ultrasonic elastic imaging	CEUS	MDCT + Unenhanced MRI	CEUS + Contrast-enhanced MRI	Normal US
**Unenhanced MRI + Contrast-enhanced MRI**	0.14 (-0.01,0.29)	0.04 (-0.04,0.12)	0.01 (-0.06,0.09)	0.02 (-0.06,0.10)	0.02 (-0.16,0.20)	0.04 (-0.05,0.13)	-0.04 (-0.14,0.05)	0.07 (-0.03,0.18)	-0.03 (-0.14,0.08)
-0.14 (-0.29,0.01)	**Normal US + CEUS + ultrasonic elastic imaging**	-0.10 (-0.23,0.03)	-0.13 (-0.26,0.01)	-0.12 (-0.25,0.01)	-0.12 (-0.27,0.03)	-0.10 (-0.22,0.02)	-0.18 (-0.33,-0.03)	-0.06 (-0.20,0.07)	-0.17 (-0.29,-0.04)
-0.04 (-0.12,0.04)	0.10 (-0.03,0.23)	**Contrast-enhanced MRI**	-0.03 (-0.08,0.03)	-0.02 (-0.06,0.02)	-0.02 (-0.18,0.14)	0.00 (-0.04,0.04)	-0.09 (-0.16,-0.01)	0.03 (-0.03,0.10)	-0.07 (-0.15,0.01)
-0.01 (-0.09,0.06)	0.13 (-0.01,0.26)	0.03 (-0.03,0.08)	**Unenhanced MRI**	0.00 (-0.06,0.06)	0.01 (-0.16,0.18)	0.03 (-0.04,0.09)	-0.06 (-0.14,0.02)	0.06 (-0.02,0.15)	-0.04 (-0.14,0.05)
-0.02 (-0.10,0.06)	0.12 (-0.01,0.25)	0.02 (-0.02,0.06)	-0.00 (-0.06,0.06)	**MDCT**	0.00 (-0.16,0.17)	0.02 (-0.03,0.08)	-0.06 (-0.14,0.02)	0.06 (-0.02,0.13)	-0.05 (-0.13,0.04)
-0.02 (-0.20,0.16)	0.12 (-0.03,0.27)	0.02 (-0.14,0.18)	-0.01 (-0.18,0.16)	-0.00 (-0.17,0.16)	**ultrasonic elastic imaging**	0.02 (-0.14,0.18)	-0.06 (-0.25,0.12)	0.05 (-0.12,0.22)	-0.05 (-0.21,0.11)
-0.04 (-0.13,0.05)	0.10 (-0.02,0.22)	-0.00 (-0.04,0.04)	-0.03 (-0.09,0.04)	-0.02 (-0.08,0.03)	-0.02 (-0.18,0.14)	**CEUS**	-0.09 (-0.17,0.00)	0.03 (-0.03,0.10)	-0.07 (-0.14,-0.00)
0.04 (-0.05,0.14)	**0.18 (0.03,0.33)**	**0.09 (0.01,0.16)**	0.06 (-0.02,0.14)	0.06 (-0.02,0.14)	0.06 (-0.12,0.25)	**0.09 (0.00,0.17)**	**MDCT + Unenhanced MRI**	0.12 (0.02,0.22)	0.02 (-0.10,0.13)
-0.07 (-0.18,0.03)	0.06 (-0.07,0.20)	-0.03 (-0.10,0.03)	-0.06 (-0.15,0.02)	-0.06 (-0.13,0.02)	-0.05 (-0.22,0.12)	-0.03 (-0.10,0.03)	**-0.12 (-0.22,-0.02)**	**CEUS + Contrast-enhanced MRI**	-0.10 (-0.20,-0.01)
0.03 (-0.08,0.14)	**0.17 (0.04,0.29)**	0.07 (-0.01,0.15)	0.04 (-0.05,0.14)	0.05 (-0.04,0.13)	0.05 (-0.11,0.21)	**0.07 (0.00,0.14)**	-0.02 (-0.13,0.10)	**0.10 (0.01,0.20)**	**Normal US**

US, ultrasound; CEUS, Contrast-enhanced ultrasound; MRI, magnetic resonance imaging; and MDCT, multi-phasic enhanced computed tomography.

The form with the background color indicates the diagnostic method used in this article;

The numerical value represents the comparison of diagnostic efficiency between two diagnostic methods and bolded data represents a meaningful difference in diagnostic performance between the two diagnostic methods.

**Table 5:** League table on NPV.

Unenhanced MRI + Contrast-enhanced MRI	Normal US + CEUS + ultrasonic elastic imaging	Contrast-enhanced MRI	Unenhanced MRI	MDCT	ultrasonic elastic imaging	CEUS	MDCT + Unenhanced MRI	CEUS + Contrast-enhanced MRI	Normal US
**Unenhanced MRI + Contrast-enhanced MRI**	-0.06 (-0.32,0.20)	-0.32 (-0.44,-0.19)	-0.28 (-0.40,-0.16)	-0.41 (-0.54,-0.28)	-0.42 (-0.69,-0.15)	-0.42 (-0.57,-0.28)	-0.42 (-0.57,-0.27)	-0.48 (-0.69,-0.27)	-0.49 (-0.67,-0.32)
0.06 (-0.20,0.32)	**Normal US + CEUS + ultrasonic elastic imaging**	-0.25 (-0.48,-0.02)	-0.22 (-0.46,0.03)	-0.35 (-0.58,-0.11)	-0.36 (-0.61,-0.11)	-0.36 (-0.57,-0.15)	-0.36 (-0.62,-0.10)	-0.42 (-0.69,-0.15)	-0.43 (-0.65,-0.22)
**0.32 (0.19,0.44)**	**0.25 (0.02,0.48)**	**Contrast-enhanced MRI**	0.03 (-0.06,0.13)	-0.09 (-0.16,-0.03)	-0.11 (-0.35,0.14)	-0.11 (-0.19,-0.03)	-0.11 (-0.24,0.02)	-0.17 (-0.33,0.00)	-0.18 (-0.31,-0.05)
**0.28 (0.16,0.40)**	0.22 (-0.03,0.46)	-0.03 (-0.13,0.06)	**Unenhanced MRI**	-0.13 (-0.23,-0.03)	-0.14 (-0.40,0.12)	-0.14 (-0.26,-0.02)	-0.14 (-0.27,-0.01)	-0.20 (-0.39,-0.01)	-0.21 (-0.37,-0.06)
**0.41 (0.28,0.54)**	**0.35 (0.11,0.58)**	**0.09 (0.03,0.16)**	**0.13 (0.03,0.23)**	**MDCT**	-0.01 (-0.26,0.24)	-0.01 (-0.10,0.08)	-0.01 (-0.14,0.11)	-0.07 (-0.25,0.11)	-0.08 (-0.22,0.05)
**0.42 (0.15,0.69)**	**0.36 (0.11,0.61)**	0.11 (-0.14,0.35)	0.14 (-0.12,0.40)	0.01 (-0.24,0.26)	**ultrasonic elastic imaging**	-0.00 (-0.23,0.23)	-0.00 (-0.27,0.27)	-0.06 (-0.35,0.23)	-0.07 (-0.31,0.16)
**0.42 (0.28,0.57)**	**0.36 (0.15,0.57)**	**0.11 (0.03,0.19)**	**0.14 (0.02,0.26)**	0.01 (-0.08,0.10)	0.00 (-0.23,0.23)	**CEUS**	0.00 (-0.14,0.15)	-0.06 (-0.22,0.11)	-0.07 (-0.17,0.03)
**0.42 (0.27,0.57)**	**0.36 (0.10,0.62)**	0.11 (-0.02,0.24)	**0.14 (0.01,0.27)**	0.01 (-0.11,0.14)	0.00 (-0.27,0.27)	-0.00 (-0.15,0.14)	**MDCT + Unenhanced MRI**	-0.06 (-0.27,0.15)	-0.07 (-0.25,0.10)
**0.48 (0.27,0.69)**	**0.42 (0.15,0.69)**	0.17 (-0.00,0.33)	**0.20 (0.01,0.39)**	0.07 (-0.11,0.25)	0.06 (-0.23,0.35)	0.06 (-0.11,0.22)	0.06 (-0.15,0.27)	**CEUS + Contrast-enhanced MRI**	-0.01 (-0.21,0.18)
**0.49 (0.32,0.67)**	**0.43 (0.22,0.65)**	**0.18 (0.05,0.31)**	**0.21 (0.06,0.37)**	0.08 (-0.05,0.22)	0.07 (-0.16,0.31)	0.07 (-0.03,0.17)	0.07 (-0.10,0.25)	0.01 (-0.18,0.21)	**Normal US**

US, ultrasound; CEUS, Contrast-enhanced ultrasound; MRI, magnetic resonance imaging; and MDCT, multi-phasic enhanced computed tomography.

The form with the background color indicates the diagnostic method used in this article;

The numerical value represents the comparison of diagnostic efficiency between two diagnostic methods and bolded data represents a meaningful difference in diagnostic performance between the two diagnostic methods.

### Unenhanced MRI + contrast-enhanced MRI

The pooled SEN, SPE, ACC, PPV, and NPV for sHCC diagnosis by Unenhanced MRI + Contrast-enhanced MRI were 96.6, 12.5, 89.1, 34.4, and 96.6, respectively. In the comparison of any two diagnostic methods, we found that compared with MDCT (OR: 0.32, 95%CI: 0.10 - 0.53), ultrasonic elastic imaging (OR: 0.49, 95%CI: 0.09 - 0.89), CEUS (OR: 0.36, 95%CI: 0.11 - 0.60), CEUS + Contrast-enhanced MRI (OR: 0.42, 95%CI: 0.07 - 0.76), and Normal US (OR: 0.66, 95%CI: 0.36 - 0.96), Unenhanced MRI + Contrast-enhanced MRI showed good diagnostic performance in SEN (**Table 6**). The combined MRI diagnostic methods had a higher NPV compared to all methods except Normal US + CEUS + ultrasonic elastic imaging (**Table 5**). However, the combined MRI diagnostic methods had poorer diagnostic performance in SEN and PPV. Compared with Normal US + CEUS + ultrasonic elastic imaging (OR: -0.53, 95%CI: -0.97 - -0.10), Contrast-enhanced MRI (OR: -0.25, 95%CI: 0.50 - -0.01), Unenhanced MRI + Contrast-enhanced MRI demonstrated poorer diagnostic performance in SPE (**Table 2**).

**Table 6:** League table on SEN.

Unenhanced MRI + Contrast-enhanced MRI	Normal US + CEUS + ultrasonic elastic imaging	Contrast-enhanced MRI	Unenhanced MRI	MDCT	ultrasonic elastic imaging	CEUS	MDCT + Unenhanced MRI	CEUS + Contrast-enhanced MRI	Normal US
**Unenhanced MRI + Contrast-enhanced MRI**	-0.22 (-0.60,0.17)	-0.22 (-0.43,-0.00)	-0.14 (-0.33,0.06)	-0.32 (-0.53,-0.10)	-0.49 (-0.89,-0.09)	-0.36 (-0.60,-0.11)	-0.21 (-0.45,0.03)	-0.42 (-0.76,-0.07)	-0.66 (-0.96,-0.36)
0.22 (-0.17,0.60)	**Normal US + CEUS + ultrasonic elastic imaging**	-0.00 (-0.33,0.32)	0.08 (-0.28,0.44)	-0.10 (-0.44,0.23)	-0.27 (-0.61,0.07)	-0.14 (-0.44,0.16)	0.01 (-0.37,0.39)	-0.20 (-0.60,0.21)	-0.44 (-0.74,-0.14)
**0.22 (0.00,0.43)**	0.00 (-0.32,0.33)	**Contrast-enhanced MRI**	0.08 (-0.09,0.25)	-0.10 (-0.21,0.01)	-0.27 (-0.62,0.08)	-0.14 (-0.26,-0.01)	0.01 (-0.20,0.22)	-0.20 (-0.47,0.07)	-0.44 (-0.66,-0.22)
0.14 (-0.06,0.33)	-0.08 (-0.44,0.28)	-0.08 (-0.25,0.09)	**Unenhanced MRI**	-0.18 (-0.35,-0.01)	-0.35 (-0.73,0.03)	-0.22 (-0.42,-0.02)	-0.07 (-0.27,0.13)	-0.28 (-0.59,0.04)	-0.52 (-0.79,-0.25)
**0.32 (0.10,0.53)**	0.10 (-0.23,0.44)	0.10 (-0.01,0.21)	**0.18 (0.01,0.35)**	**MDCT**	-0.17 (-0.53,0.18)	-0.04 (-0.18,0.11)	0.11 (-0.09,0.31)	-0.10 (-0.38,0.19)	-0.34 (-0.58,-0.11)
**0.49 (0.09,0.89)**	0.27 (-0.07,0.61)	0.27 (-0.08,0.62)	0.35 (-0.03,0.73)	0.17 (-0.18,0.53)	**ultrasonic elastic imaging**	0.13 (-0.19,0.46)	0.28 (-0.12,0.68)	0.07 (-0.35,0.50)	-0.17 (-0.50,0.15)
**0.36 (0.11,0.60)**	0.14 (-0.16,0.44)	**0.14 (0.01,0.26)**	**0.22 (0.02,0.42)**	0.04 (-0.11,0.18)	-0.13 (-0.46,0.19)	**CEUS**	0.15 (-0.09,0.38)	-0.06 (-0.33,0.21)	-0.31 (-0.49,-0.12)
0.21 (-0.03,0.45)	-0.01 (-0.39,0.37)	-0.01 (-0.22,0.20)	0.07 (-0.13,0.27)	-0.11 (-0.31,0.09)	-0.28 (-0.68,0.12)	-0.15 (-0.38,0.09)	**MDCT + Unenhanced MRI**	-0.21 (-0.54,0.13)	-0.45 (-0.75,-0.16)
**0.42 (0.07,0.76)**	0.20 (-0.21,0.60)	0.20 (-0.07,0.47)	0.28 (-0.04,0.59)	0.10 (-0.19,0.38)	-0.07 (-0.50,0.35)	0.06 (-0.21,0.33)	0.21 (-0.13,0.54)	**CEUS + Contrast-enhanced MRI**	-0.25 (-0.57,0.08)
**0.66 (0.36,0.96)**	0.44 (0.14,0.74)	**0.44 (0.22,0.66)**	**0.52 (0.25,0.79)**	**0.34 (0.11,0.58)**	0.17 (-0.15,0.50)	**0.31 (0.12,0.49)**	**0.45 (0.16,0.75)**	0.25 (-0.08,0.57)	**Normal US**

US, ultrasound; CEUS, Contrast-enhanced ultrasound; MRI, magnetic resonance imaging; and MDCT, multi-phasic enhanced computed tomography.

The form with the background color indicates the diagnostic method used in this article;

The numerical value represents the comparison of diagnostic efficiency between two diagnostic methods and bolded data represents a meaningful difference in diagnostic performance between the two diagnostic methods.

### Contrast-enhanced MRI

Contrast-enhanced MRI performed better overall in individual imaging methods. The pooled SEN, SPE, ACC, PPV, and NPV of sHCC diagnosed by Contrast-enhanced MRI were 66, 55.5, 67.9, 6.44, and 66.5, respectively. Compared with CEUS (OR: 0.14, 95%CI: 0.01 - 0.26) and Normal US (OR: 0.44, 95%CI: 0.22 - 0.66), Contrast-enhanced exhibited higher SEN (**Table 6**). Compared with MDCT + Unenhanced MRI (OR: 0.29, 95%CI: 0.07 - 0.52), Contrast-enhanced MRI showed higher SPE (**Table 2**). It showed higher ACC compared to Normal US (OR: 0.18, 95%CI: 0.03 - 0.33) (**Table 3**). It showed higher PPV compared to MDCT + Unenhanced MRI (OR: 0.09, 95%CI: 0.01 - 0.16) (**Table 4**). Contrast-enhanced MRI exhibited higher NPV compared to MDCT (OR: 0.09, 95%CI: 0.03 - 0.16), CEUS (OR: 0.11, 95%CI: 0.03 - 0.19), and Normal US (OR: 0.18, 95%CI: 0.05 - 0.31) (**Table 5**).

### Analysis of heterogeneity and sensitivity analyses

Significant heterogeneity was found in our experiment (*P* < 0.01, I^2^ = 99%). The main relevant diagnostic methods for the loop arms that did not pass the zero-effect line were found by the loop inconsistency results to be MDCT, Unenhanced MRI, MDCT + Unenhanced MRI, and Unenhanced MRI + Contrast-enhanced MRI. Related studies are the R. Golfieri et al. (33) and Thomas Sersté et al. (40) Preliminary heterogeneity analysis did not reveal any significant sources of heterogeneity. For further discussion, this study found that removing the experimental data of Thomas Sersté et al. (40) the SEN, SPE inconsistency test was not significant (SEN: *P* = 0.16; SPE: *P* = 0.88), at which point the SEN, SPE consistency assumption could be accepted. The SUCRA reordering for SEN and SPE is shown in **Table 7**. It was found that the literature data of Thomas Sersté et al. (41) had less impact on the overall diagnostic efficacy ranking. There were no contradictions between the results of the sensitivity analysis and the results of the initial analysis.

**Table 7:** SUCRA ranking of sHCC diagnostic methods after excluding target experiments.

DIAGNOSTIC METHODS	SUCRA values				
	SEN	SPE	ACC	PPV	NPV
ultrasonic elastic imaging	18.9	66.1	33.2	47.8	35.4
Normal US	1.9	62.8	9.4	17.6	12.3
Normal US + CEUS + ultrasonic elastic imaging	60.2	94.7	93.6	94.4	91.7
CEUS	34.3	61.3	32.6	63.2	33.6
CEUS + Contrast-enhanced MRI	26.8	74.8	24.9	81.6	19.2
Contrast-enhanced MRI	61.6	51.8	67.9	64.4	66.5
MDCT	37.8	52.9	38.4	43.3	38.2
Unenhanced MRI	76.9	20.8	70.5	41.9	73.3
MDCT + Unenhanced MRI	85.7	1.6	40.5	11.4	33.2
Unenhanced MRI + Contrast-enhanced MRI	95.8	13.4	89.1	34.4	96.6

The number with the background color indicates the highest SUCRA;

SUCRA, the area under the cumulative ranking curve; US, ultrasound; CEUS, Contrast-enhanced ultrasound; MRI, magnetic resonance imaging; and MDCT, multi-phasic enhanced computed tomography.

## Discussion

To compare the diagnostic value of various sHCC-related diagnostic imaging methods, 16 high-quality original studies were included, which included data from 2447 sHCC patients. The included literature had good methodological quality and there was no obvious publication bias, but some of them had inconsistency problems and high heterogeneity.

In our study, we focused on finding that the combined ultrasound diagnostic methods of Normal US + CEUS + ultrasonic elastic imaging had the highest SPE (92.9), ACC (93.6), and PPV (94.4). NPV (91.7) ranked second only to Unenhanced MRI + Contrast-enhanced MRI (96.0). Its SUCRA value was far higher than that of Normal US, ultrasonic elastic imaging, and CEUS, which was the best imaging choice for the diagnosis of sHCC. In this experiment, ultrasonic elastic imaging and Normal US showed high SPE (ultrasonic elastic imaging: SPE = 64.7; Normal US: SPE = 62.1), and can be used as first-line screening diagnosis for liver nodules. Considering factors such as cost-effectiveness, the first-line assessment of sHCC screening is usually performed using Normal US. It can improve overall diagnostic accuracy while reducing the incidence of FN results in subsequent imaging modalities. The AUROC for CEUS imaging in this study was 0.94 (95% CI, 0.92 - 0.96). A study by Pei-Li Fan et al. (48) found a significant difference in the arterial enhancement phase of sHCC and high-grade dysplastic nodule when diagnosed by CEUS. The CEUS characteristics of sHCC are related to its blood supply and physiological characteristics, mainly manifested as rapid and significant enhancement in the arterial phase, and low echo “washout” in the portal venous phase and delayed phase. The rapid growth of cancer beyond physiological control leads to the formation of a large number of irregular and disordered neovascularization inside, with poor differentiation. After the contrast agent is injected into the body, it quickly reaches the liver tissue through systemic circulation and is diluted by portal venous blood. However, sHCC supplied solely by the hepatic artery has a stronger echo enhancement compared to normal liver tissue with diluted contrast agent concentration. During the venous phase, contrast agents mainly exist in the venous blood with blood circulation, and the hepatic artery supplying blood to the mass contains less contrast agent, exhibiting a relatively low echo “washout” phenomenon. The new contrast agent Sonazoid provides a new approach for the diagnosis of sHCC ultrasound contrast with characteristic Kupffer phase manifestations. CEUS-specific typical imaging manifestations could improve the diagnostic SPE of CEUS. The misdiagnosis rate could be significantly reduced. The progression of portal hypertension was strongly correlated with the severity of liver disease (49, 50). Peng Wang et al. (51) the measurement of hepatosplenic stiffness by two-dimensional elastography was positively correlated with portal venous pressure in liver disease patients and the indirect signs can assist in the diagnosis of sHCC. If a nodule is detected at the time of sHCC screening, a comprehensive and accurate examination with CEUS and/or ultrasonic elastic imaging testing may be considered immediately following the regular ultrasound screening to improve diagnostic SEN and resolve the problem on the same visit. Although the combined MRI diagnostic methods had the highest SEN (96.6) and NPV (96.6), with significantly higher SUCRA values than Unenhanced MRI and Contrast-enhanced MRI, it exhibited extremely poor SPE (12.5) and PPV (34.4). Firstly, excessively low SPE and PPV may lead to biased risk assessment of patients by physicians or decision-makers, thus affecting the selection and implementation of treatment options. Secondly, it may also lead to the normal population receiving unnecessary interventions and wasting medical resources. It also leads to greater trauma to the patient’s psyche. The development of sHCC is a multistep process involving chronic liver disease, cirrhosis, and precancerous lesions, and those with positive initial screening results must undergo more expensive, accurate, and invasive biopsies or imaging tests for further clarification and long-term monitoring (52). Close surveillance and early diagnosis and treatment of these lesions may improve the survival of patients with sHCC (53). Currently, there is a large base of patients in need of surveillance, and ultrasound offers convenience, immediate diagnosis, no contraindications to diagnosis, no radiation damage, and low economic costs. Compared to MRI regional imbalance in economic distribution, dependence on technicians, need for more patient cooperation, heavy economic burden and other circumstances. We suggest that in clinical practice, ultrasound and its combined diagnostics should first be used for screening of sHCC in combination with the patient’s general condition and clinical indications. Later on, combined examination with MRI is the preferred technique for further diagnosis in the screened abnormal population. Although the combined diagnosis of multiple imaging methods showed better diagnostic performance, considering the possible problems of overmedication and heavier economic costs of combined diagnosis, clinicians should make choices with full consideration of the patient’s condition, economy, time, and other specifics in the actual decision-making.

In individual imaging methods, Contrast-enhanced MRI demonstrated better diagnostic value in all aspects. A retrospective study by Kui Sun et al. (54) found that a radiomics joint feature model based on multiphase-enhanced MRI revealed small hepatocellular pre-cancerous loss, with an area under the receiver operating characteristic curve (AUROC) of subjects of 0.93 (95% CI: 0.85 - 1.00), and our study was consistent with its study for Contrast-enhanced MRI imaging with an AUROC of 0.92 (95% CI: 0.89 - 0.94). The characteristic imaging manifestation of sHCC in Contrast-enhanced MRI is initial edge enhancement during the arterial phase, followed by gradual filling towards the center in dynamic contrast-enhanced imaging. Contrast-enhanced MRI has imaging value in distinguishing precancerous lesions from sHCC. Microvascular invasion (MVI) is one of the independent predictors of sHCC relapses and poorer prognosis, which may suggest poor differentiation, aggressiveness, or the presence of highly malignant behavior in sHCC (25, 55). Xinxin Wang et al. (56) measured MVI in sHCC patients by Gd-EOB-DTPA-enhanced magnetic resonance hepatobiliary phase imaging, founding that mean lesion border index and edge gray change values of the MVI-positive group were significantly lower than those of the MVI-negative group. Enhanced magnetic resonance not only provides an accurate diagnosis of sHCC, but its quantitative analysis of MVI also reveals early malignant differentiation of cancerous nodules, making early intervention in the treatment of the mass a practically valuable imaging method in clinical selection.

The apparent heterogeneity of the data from the SEN and SPE studies in this study may mean that valuable indirect comparisons cannot be made (57). Sensitivity analysis by article-by-article deletion found the source of inconsistency to be in the data from the study by Thomas Sersté et al. (40) Re-performing effect synthesis by removing the study found little change in the results after exclusion. This proved that there were no significant bias factors related to intervention effectiveness in our experiment, and also demonstrated that our results were robust and reliable. Six studies in the article were at high risk for methodological quality, but the results of heterogeneity analysis found that the heterogeneity of QUADAS-2 is not significant (SEN: *P* = 0.35; SPE: *P* = 0.47). It indicates that the validation bias caused by not using a uniform reference standard to determine the disease status of sHCC had a small impact on the study results in this study. Although the existence of publication bias is inevitable in the implementation of the meta-analysis, the funnel plot results indicate that publication bias is not significant in this study.

Our findings support several recent practice guidelines and also provide comparative new data that can influence clinical practice. Our results agree with that of a prior imaging meta-analysis evaluating CEUS and contrast-enhanced computed tomography in diagnosing sHCC by Jiasheng Huang et al. (58) The analysis of this study found that the AUROC for contrast-enhanced computed tomography and CEUS were 0.89 and 0.91, respectively. And our experimental results showed that the AUROC for MDCT and CEUS were 0.87 (0.83 - 0.91) and 0.94 (0.92 - 0.96), respectively. Another meta-analysis on the imaging diagnosis of sHCC elaborated on the advantages of CEUS (59). However, these meta-analysis studies didn’t perform a comprehensive analysis of the ability of arbitrary imaging diagnosis regarding sHCC. This network meta-analysis included the comprehensive results obtained from US, MDCT, and MRI for the first time, which are commonly used noninvasive imaging methods in clinical practice, and assessed the relative effectiveness between diagnostic measures by combining direct and indirect evidence to compare and quantify different diagnostic measures, which solved the problem of the lack of direct comparative evidence. Meanwhile, network meta-analysis improves statistical efficacy and accuracy, and readers can rationally choose the diagnostic methods based on clinical practice experience and patients’ actual conditions. However, this study still has limitations: first, this experiment included fewer original studies, and some diagnostic methods have been less studied in the original literature and clinical practice, which may lead to inaccurate estimation of heterogeneity and affect the conclusions of meta-analysis, limiting the generalizability of the findings. Secondly, sHCC and early hepatocellular carcinoma are not identical in the histologic definition, and whether the early imaging diagnosis of sHCC can accurately match the results of the definition of early hepatocellular carcinoma pathophysiology still requires in-depth research by posterity (60). Third, there was significant heterogeneity and inconsistency among the studies in this trial. It may be due to differences in the baseline characteristics of patients or the design methods of the study. Finally, the original literature we included did not strictly require 3.0 cm for the selection of sHCC, and some studies with sHCC diameters < 2.0 cm were included at the same time, which may lead to bias such as lower SEN in the combined results. Based on these limitations, future studies should be implemented based on continued collection of diagnostic accuracy data, strict definition of inclusion criteria, and large prospective trials are recommended to explore the possibility of achieving higher levels of accuracy by combining methods.

## Data Availability

The original contributions presented in the study are included in the article/**Supplementary Material**. Further inquiries can be directed to the corresponding authors.

## Genertaive AI statement

The authors declare that no Gen AI was used in the creation of this manuscript.
