# Supplementary figures and images for: Application value of different imaging methods in the early diagnosis of small hepatocellular carcinoma: a network meta-analysis
Source: Front Oncol. 2025 Jan 14;14:1510296. doi: 10.3389/fonc.2024.1510296 (PMC11772129; doi:10.3389/fonc.2024.1510296)

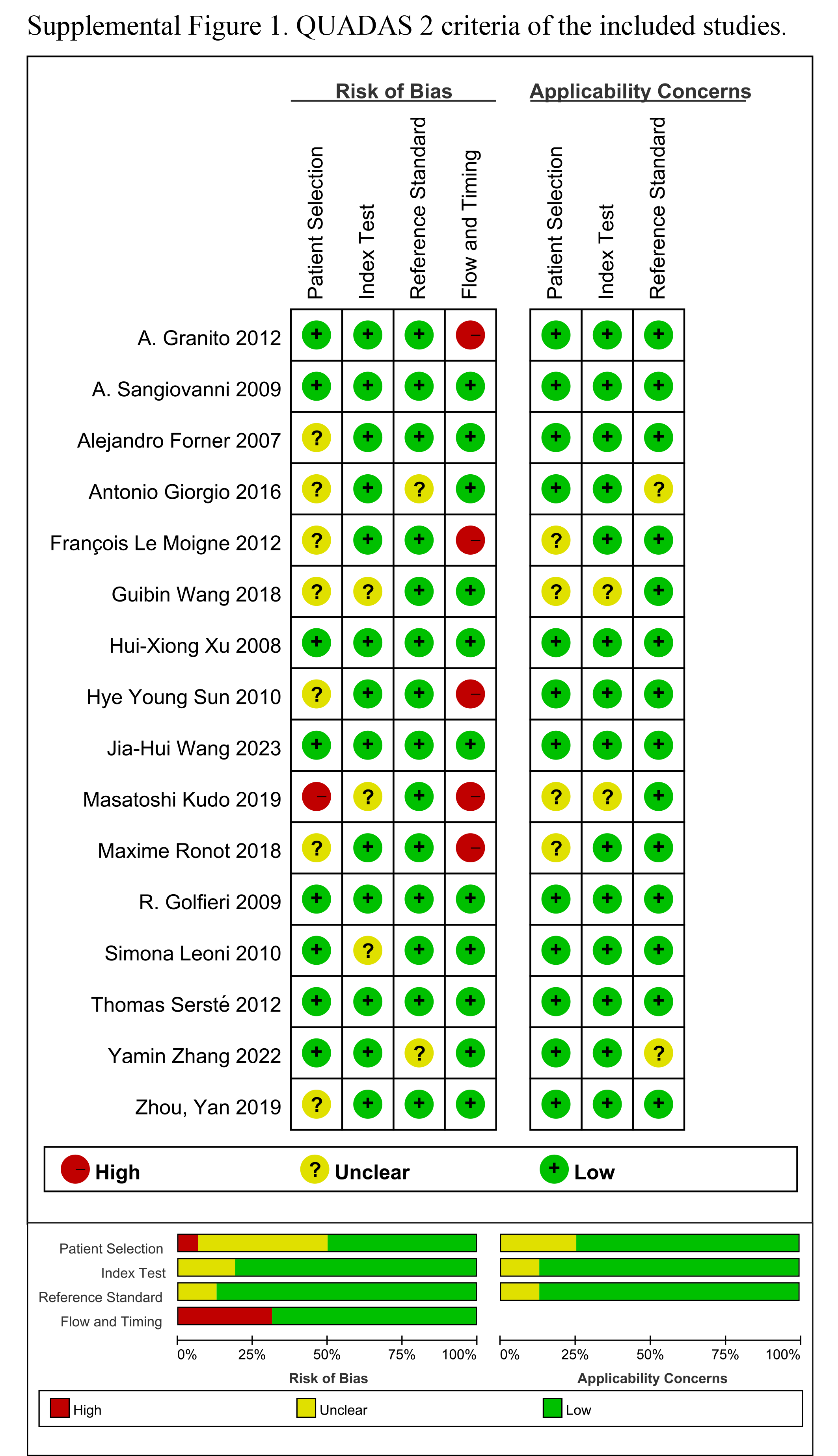

Supplement: Supplementary file 1 [file Image1.tif]

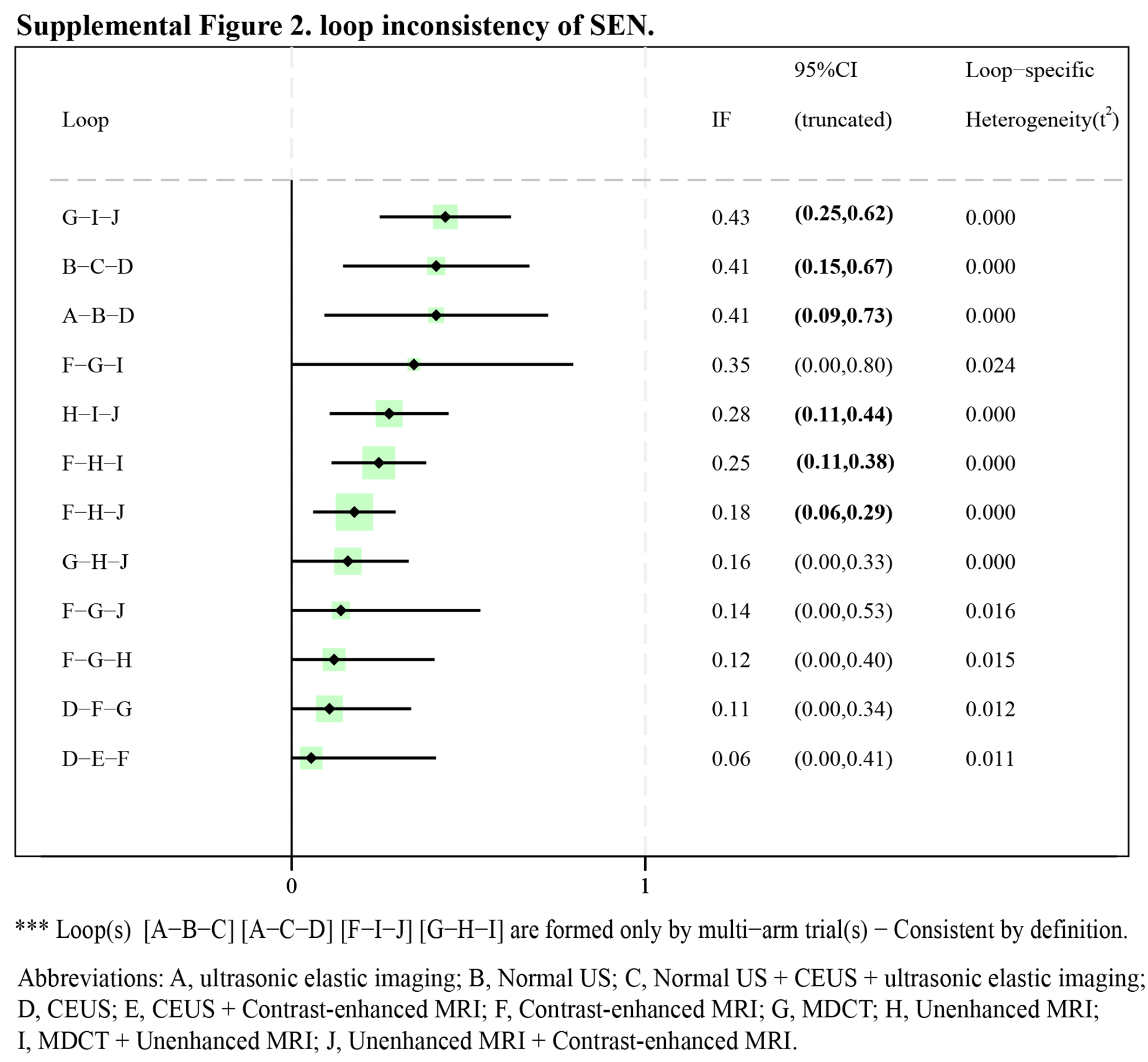

Supplement: Supplementary file 2 [file Image2.tif]

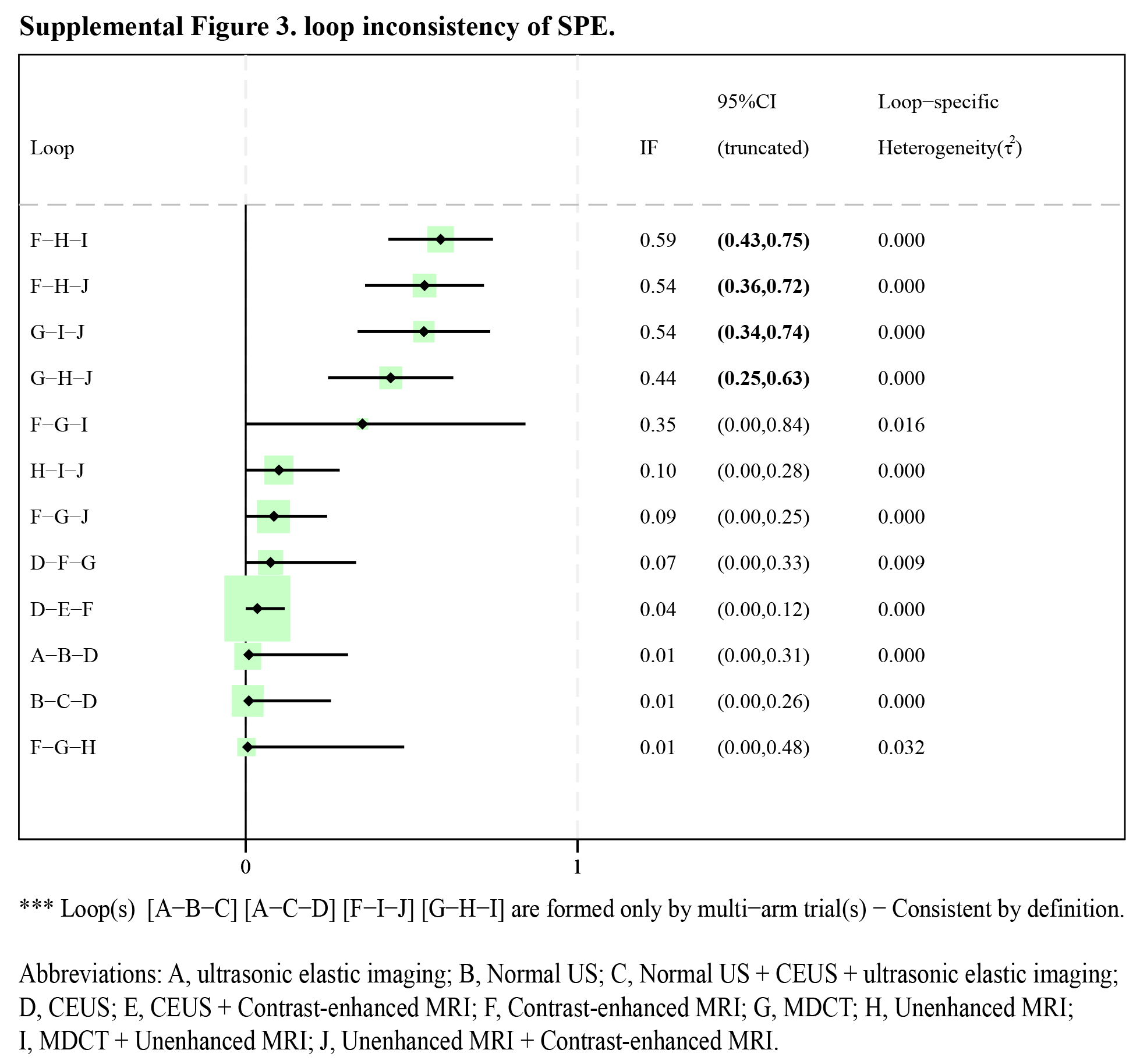

Supplement: Supplementary file 3 [file Image3.tif]
