# Supplementary material for: Application value of different imaging methods in the early diagnosis of small hepatocellular carcinoma: a network meta-analysis
Source: Front Oncol. 2025 Jan 14;14:1510296. doi: 10.3389/fonc.2024.1510296 (PMC11772129; doi:10.3389/fonc.2024.1510296)
Supplement: Supplementary file 5 [file Table1.docx]

| **Supplemental Table 1. Characteristics of the trials.** | | | | | | | | | | |
| --- | --- | --- | --- | --- | --- | --- | --- | --- | --- | --- |
| **Author** | **Year** | **Country** | **Type of Study** | **NO. of patients** | **NO. of nodules** | **NO. of sHCC** | **age** | **Gender**  **(male/female)** | **Reference standard** | **Imaging modality** |
| Alejandro Forner(31) | 2007 | Spain | Prospective | 89 | 89 | 60 | 65 | 53/36 | fine-needle biopsy | Contrast-enhanced MRI |
|  |  |  |  |  |  |  |  |  |  | CEUS |
| Antonio Giorgio(32) | 2016 | Italy | Prospective | 229 | 229 | 199 | 69 | 137/92 | percutaneous US-guided needle biopsy | Contrast-enhanced MRI |
|  |  |  |  |  |  |  |  |  |  | CEUS |
| R. Golfieri(33) | 2009 | Italy | Prospective | 63 | 123 | 87 | 62.8 | 53/10 | surgical specimens | Unenhanced MRI |
|  |  |  |  |  |  |  |  |  |  | MDCT |
|  |  |  |  |  |  |  |  |  |  | Contrast-enhanced MRI |
|  |  |  |  |  |  |  |  |  |  | MDCT + Unenhanced MRI |
|  |  |  |  |  |  |  |  |  |  | Unenhanced MRI + Contrast-enhanced MRI |
| A. Granito(34) | 2012 | America | Prospective | 33 | 41 | 38 | 70 | 25/8 | percutaneous US-guided needle biopsy or US/CT along with AFP every 3 months | CEUS |
|  |  |  |  |  |  |  |  |  |  | MDCT |
|  |  |  |  |  |  |  |  |  |  | Contrast-enhanced MRI |
| Masatoshi Kudo(35) | 2019 | Japan | Prospective | 656 | 623 | 54 | 67.5 | 268/354 | liver biopsy or radiologically | CEUS |
|  |  |  |  |  |  |  |  |  |  | Normal US |
| François Le Moigne(36) | 2012 | France | Prospective | 62 | 82 | 66 | 63.6 | 53/9 | surgical resection, transplantation or  biopsy | Unenhanced MRI |
|  |  |  |  |  |  |  |  |  |  | Unenhanced MRI + Contrast-enhanced MRI |
| Simona Leoni(37) | 2010 | Italy | Prospective | 60 | 75 | 44 | 65.2 | 52/8 | biopsy | CEUS |
|  |  |  |  |  |  |  |  |  |  | MDCT |
|  |  |  |  |  |  |  |  |  |  | Contrast-enhanced MRI |
| Maxime Ronot(38) | 2018 | France | Prospective | 422 | 595 | NR | 61.7 | 343/69 | liver resection, liver transplantation or biopsy | MDCT |
|  |  |  |  |  |  |  |  |  |  | Contrast-enhanced MRI |

| **Table 1. Continued characteristics of the trials.** | | | | | | | | | | |
| --- | --- | --- | --- | --- | --- | --- | --- | --- | --- | --- |
| **Author** | **Year** | **Country** | **Type of Study** | **NO. of patients** | **NO. of nodules** | **NO. of sHCC** | **age** | **Gender**  **(male/female)** | **Reference standard** | **Imaging modality** |
| A. Sangiovanni(39) | 2009 | Italy | Prospective | 64 | 67 | 44 | 65 | 47/17 | fine-needle biopsy | CEUS |
|  |  |  |  |  |  |  |  |  |  | MDCT |
|  |  |  |  |  |  |  |  |  |  | Contrast-enhanced MRI |
| Thomas Sersté(40) | 2012 | France | Prospective | 74 | 74 | 47 | 60 | 58/16 | biopsy | MDCT |
|  |  |  |  |  |  |  |  |  |  | Unenhanced MRI |
|  |  |  |  |  |  |  |  |  |  | MDCT + Unenhanced MRI |
| Hye Young Sun(41) | 2010 | Korea | Retrospective | 69 | 97 | 44 | 55.8 | 56/13 | surgical resection or percutaneous biopsy | MDCT |
|  |  |  |  |  |  |  |  |  |  | Contrast-enhanced MRI |
| Guibin Wang(42) | 2018 | China | Retrospective | 300 | 300 | 170 | 43.46 | 186/114 | surgical resection or biopsy | MDCT |
|  |  |  |  |  |  |  |  |  |  | Contrast-enhanced MRI |
| Huixiong Xu(43) | 2008 | China | Prospective | 104 | 104 | 49 | 48 | 81/23 | biopsy | Normal US |
|  |  |  |  |  |  |  |  |  |  | CEUS |
| Yamin Zhang(44) | 2022 | China | Prospective | 100 | 152 | 110 | 45.24 | 64/36 | biopsy | Normal US |
|  |  |  |  |  |  |  |  |  |  | CEUS |
|  |  |  |  |  |  |  |  |  |  | ultrasonic elastic imaging |
|  |  |  |  |  |  |  |  |  |  | Normal US + CEUS + ultrasonic elastic imaging |
| Zhou, Yan(45) | 2019 | China | Retrospective | 98 | 116 | 89 | 58.13 | 67/31 | pathologic biopsy | CEUS |
|  |  |  |  |  |  |  |  |  |  | Contrast-enhanced MRI |
|  |  |  |  |  |  |  |  |  |  | CEUS + Contrast-enhanced MRI |
| Jia-Hui Wang(46) | 2023 | China | Retrospective | 204 | 237 | 166 | 54 | 163/41 | pathologic biopsy | Unenhanced MRI |
|  |  |  |  |  |  |  |  |  |  | Contrast-enhanced MRI |
| Abbreviations: MRI, magnetic resonance imaging; CEUS, Contrast-enhanced ultrasound; US, ultrasound; MDCT, multi-phasic enhanced computed tomography; CT, computed tomography; AFP, alpha fetoprotein; and NR, not report. | | | | | | | | | | |
